# Joy to the World

**Published:** 1875-02

**Authors:** 


### JOY TO THE WORLD

Of old Bachelors, of woe-begone widowers, and that most unfortunate class of husbands, whose wives either can't or won't sew on their buttons, for, without needle or thread, a button can be put on in less than half a minute, which will never come off as long as the garment can be worn. For this great boon the public are indebted to

### BARNEY & CO.,

MANUFACTURERS,

### 234 Broadway, New York, Room 22,

Where Agents are wanted who are looking for an easy, money-making business. Exclusive territory will be assigned to enterprising persons who wish to employ Sub-Agents. The invention is styled

### BARNEY'S

### CURVILINEAL SPRING STEEL

### BUTTON HOLDER,

FOR LADIES' AND GENTLEMEN'S CLOTHING AND BUTTONED BOOTS.

It is believed to be the most durable, economical and universally approved invention now in use.

For sale by agents in all the principal cities, not farmed out, at 25 cents a box, containing 25 Button Holders. A box holding a dozen small boxes is sold for $1; hence each person investing one dollar, will have three returned. It will be thus seen that the risk to sub-agents is small, and the net profits two hundred per cent.

These Button Fasteners are not only needed in every family, but in every

### BACHELOR'S HALL,

By all Students away from home, at school, Academy, College or University.

Single boxes will be sent by mail, post paid, for 25 cents; or the large boxes for one dollar.

Persons desiring exclusive territory for cities and large towns, would do well to apply without delay.

